# Observing Social Exclusion Leads to Dehumanizing the Victim

**DOI:** 10.3389/fpsyg.2015.01815

**Published:** 2015-11-24

**Authors:** Yeong O. Park, Sang H. Park

**Affiliations:** Department of Psychology, Chungbuk National University, Cheongju, South Korea

**Keywords:** ostracism, exclusion, dehumanization, mind perception, social perception

## Abstract

We hypothesized that observing social exclusion would influence observers’ judgments of the humanness of its victims and perpetrators. Specifically, we speculated that people would attribute victims and perpetrators to lower and higher mental capacities, respectively. Participants observed a simulated computer-based ball tossing game in which one of the players was either ostracized or not. They then rated the game players on traits associated with two dimensions of humanness, namely Human Nature (HN) and Human Uniqueness (HU). Overall, participants who witnessed an exclusion game judged the victim as less human on both domains compared to one of the perpetrators as well as to a player in the control condition. The perpetrator was attributed higher HN, but not significantly higher HU, compared to the control player. In addition, the less HN attributes a target was assigned, the more she was expected to be vulnerable to exploitation. On most of the other measures of target impression, however, the victim was rated more favorably than the perpetrator. The findings imply that social exclusion victims are often subtly derogated compared to the perpetrators, even while they are also more positively evaluated otherwise.

## Introduction

Ostracism is a social phenomenon in which a person is being excluded and ignored by others (Williams, 2007). It has detrimental effects on the victims (Olweus, 1993; Williams, 2001; Baumeister et al., 2007), sometimes resulting in extreme and tragic consequences such as school shootings or suicides. In social contexts where ostracism occurs, third party observers often play the role of passive bystanders (Howard et al., 2014). Such non-intervention by observers may be interpreted by the perpetrators as implicit condoning of their malicious actions and thus can effectively lead to further reinforcement of those actions (Nishina and Juvonen, 2005). Therefore, understanding the psychological factors that keep observers from intervening in ostracism may offer hints about how to better regulate ostracism. In the current research, we focused on observers’ perception of victims and perpetrators of social exclusion. Specifically, we examined the extent to which observers perceive mental qualities that distinguish human beings from other animals or objects (Haslam et al., 2005) in the ostracized and the ostracizing. By demonstrating that observers are less likely to ascribe human qualities to victims of social exclusion and thereby subtly derogate them, we aim to offer one explanation of why observers often neglect to intervene in and even justify social exclusion.

### Ostracism and its Observers

Ostracism often occurs when there are others around, and these other people can play distinct and significant roles in ostracism. Some may actively assist the perpetrator, others may defend the victim, and still others may just keep acting as passive bystanders (Olweus, 1993; Salmivalli, 2010). Studies have shown that observers’ appropriate reactions to ostracism can be effective in reducing its negative impacts on the victim (Salmivalli et al., 2011). For example, observers’ active intervention can give victims psychological benefits such as decrease in negative emotions and boost in self-esteem (Sainio et al., 2011), while passively witnessing the victim’s adversity does not provide such help (Nishina and Juvonen, 2005). However, observers only rarely actively help the victim (Salmivalli and Voeten, 2004). There are several reasons for this: For one thing, people are influenced by others around them (Bandura, 1977), so if there is no one intervening, observers may not take the situation as serious enough for attention (Chekroun and Brauer, 2002; Teräsahjo and Salmivalli, 2003). Or observers may not feel personally responsible for defending the victims (Latané and Darley, 1968) and instead expect others to take actions. They may also decide to stay silent for fear of retaliation from the perpetrator (Thornberg, 2007) or because of low expected self-efficacy of their intervention (Hazler, 1996; Lodge and Frydenberg, 2005).

Even at the same time as observers generally report sympathy toward ostracism victims, they often exhibit negative feelings at the victims. Rigby (1996) noted that although many bystanders reported anti-bullying attitudes, nearly half of the sample indicated negative attitudes not toward the perpetrators, but toward the victims. According to more recent research, observers with biased perceptions of the victim direct the blame and responsibility for the situation at the victim (Schuster, 2001; Teräsahjo and Salmivalli, 2003): When people become aware of the victim’s previous history of victimization, they are more likely to put the blame or responsibility for the ostracism situation on the victim (Schuster, 2001). Accordingly, once someone is perceived as a victim, people would think that the victim somehow deserves their plight and be indifferent to the victim’s suffering, which may contribute to even further exclusion. Given the reasons for observers’ passivity after witnessing an exclusion incident, it is probable that they would form impressions of the victim’s characteristics that can offer explanations about why the exclusion is taking place, and such impressions can be unfavorable toward the victims.

### Ostracism and Denial of Human Values

One possibility that observers of ostracism would treat the victim is to neglect to perceive the target’s aspects as a human being. Kelman (1976) defined dehumanization as “denying a person’s characteristics as an individual, independent human and his interconnectedness as part of a social network.” As such, dehumanizing entails deprivation of the rights to be treated inside the moral circle (Bandura, 1999). Although extreme dehumanization—whereby a human being is not perceived as one—would be limited to extreme cases, researchers have demonstrated how more subtle forms of dehumanization can emerge in various situations. Recent years have witnessed several different dimensions of human qualities put forth; a dearth of any of these would imply that the target being perceived is not entirely worthy of being treated as a human being. These dimensions include agency and experience (Gray et al., 2007), competence, warmth, and morality (Fiske et al., 2002; Leach et al., 2007), and secondary emotions (Leyens et al., 2000).

In the current research, we employ the model by Haslam et al. (2005). Haslam et al. (2005) argued that to be treated as a human being, assumption of mental capacities that are largely distinct from one another is required. Specifically, building on previous conceptualizations of dehumanization, they suggested two dimensions of human characteristics that differentiate human beings from machines (*Human Nature*; HN) and from animals (*Human Uniqueness*; HU), respectively. HN traits are deeply rooted, unchanging, and inherent human characteristics, associated with social warmth, emotionality, and cognitive openness. HU traits, on the other hand, are mental dimensions traits that represent cognitive capability, civility and moral sensibility, acquired through cognitive maturation (Haslam et al., 2005; Haslam, 2006). Using this framework, Haslam and their colleagues have shown that people are less likely to attribute human qualities to others versus the self (Haslam et al., 2005) and to outgroups versus the ingroup (Bain et al., 2009). More recently, Yang et al. (2015) demonstrated that dehumanization on these two dimensions can happen even to the self, when the self is thought to be lacking in power.

Ostracism ruins people’s connection to their social community. It leads to perceived loss of what they had essentially in common with others, namely the human value. Bastian and Haslam (2010) demonstrated that those involved in ostracism (as the victim or as the perpetrator) perceive themselves and the other person differently on these dimensions. In their research, victims of social exclusion perceived themselves as lower on humanness (Haslam et al., 2005) compared to those uninvolved in ostracism. They not only attributed traits that define humanness to themselves less than controls did of themselves, but also expected that they would be seen as having less human traits by a third party (Bastian and Haslam, 2010). Perpetrators also perceived the victims (in addition to themselves) as less human (Bastian et al., 2013). These findings demonstrated how social disconnection is related to perception of human values.

We suspected that by dehumanizing ostracism victims, observers cannot only explain why the victims were excluded, but also justify their indifference and inaction toward them. Research that examined causes of observers’ inaction in ostracism focused mostly on bystander effect (Latané and Darley, 1970; Hazler, 1996; Teräsahjo and Salmivalli, 2003; Lodge and Frydenberg, 2005; Pozzoli and Gini, 2010), and it has been rarely investigated whether and how observers’ judgment of the victim’s (and the perpetrator’s) human qualities can be affected by exclusion status.

### Hypothesis and Study Overview

Although observers of social exclusion in general report pro-victim attitudes (Salmivalli and Voeten, 2004), they often harbor negative beliefs toward the victims at the same time, that the victim is deviant from non-victims and/or deserves the adversity (Schuster, 2001). In the current study, we investigated how witnesses of ostracism perceive the victim as well as the perpetrators of ostracism in terms of human qualities. We hypothesized that observers would depreciate the victim’s human qualities (i.e., human nature and human uniqueness) in subtle ways. Such victim derogation can serve at least two functions: to explain why ostracism happened (so that they can make sense of the associated injustice) as well as to justify their own inaction (so that they would feel less discomfort). To demonstrate this, we examined how observers would evaluate the victim on Haslam et al. (2005) HN and HU traits. Additionally, we examined how dehumanization of exclusion victim, if it occurs, could account for observers’ expectations about the victim’s lower social functioning; to be more specific, of being exploited by others. We expected that to the extent that the victim was perceived as lacking in human qualities, they would be more likely to be seen as vulnerable to being taken advantage of by others.

What about the perpetrators—would they be assigned relatively higher human qualities than the victims? Past research has demonstrated that ostracism perpetrators are dehumanized by both the victims and the perpetrators themselves (Bastian et al., 2013) but it was open to question how observers would see them compared to both the victims and to those uninvolved in ostracism.

Lastly, for exploratory purposes, we also measured and compared several other constructs studied in the mind perception and the dehumanization literature (Agency and Experience: Gray et al., 2007; Competence, Warmth, and Morality: Fiske et al., 2007; Leach et al., 2007) as well as Big 5 personality trait dimensions (John and Srivastava, 1999), to see whether we would observe patterns of dehumanization or victim derogation on these aspects in a similar fashion as on HN and HU. Specifically, we examined whether our hypothesized dehumanization of the exclusion victim would generalize to these other measurements of mind perception. Because it was for mainly exploratory reasons that we included these measures, we left as an open question how social exclusion would affect participants’ perception of the victim (and the perpetrator) on these dimensions.

## Materials and Methods

### Participants

Two hundred and eighteen undergraduate students taking psychology courses in a large South Korean public university (99 women, age *M* = 21.5, SD = 2.1) took part in the study for partial course credit. This study was reviewed and approved by the departmental Institutional Review Board (which operated in lieu of the not-yet-established university-level IRB). The study was conducted in compliance with the ethical standards of APA in the treatment of the human subject sample.

### Procedure

Participants were seated in cubicles individually. After they signed the written consent form, participants were told that the purpose of this study was to investigate if people can guess others’ psychological characteristics solely by observing their online activities. And then they watched an alleged “recorded session” of an online ball-tossing game. The clip was actually a simulated session of Cyberball, a game paradigm used in studies on psychological effects of ostracism (Williams et al., 2000). In the original Cyberball procedure, participants play the game with two or three other “players” (who actually are computer-controlled) and are made to experience being excluded or not, through manipulation of the number of ball tosses. In this study, we modified the Cyberball paradigm so that all three players were controlled by computer program and participants simply watched the game as an observer. Also unlike in the original three-player Cyberball (in which the three players were positioned in an upside-down triangular formation), the middle player was placed at the top of the triangle to prevent participants from spontaneously identifying with that person (Libby et al., 2009). Participants were randomly assigned to one of two conditions (Control or Exclusion) and watched the assigned game. In the Control game, each player received the ball one third of the time from other players, while in the Exclusion game one of the three players (randomly chosen to be either the left or the right player) was tossed the ball only twice and was ignored by the other players for the rest of the game. The total throws in each game were 30. Throughout the session, faces described as the players’ (all female) were displayed next to the corresponding virtual ball-tossers. Participants then rated their impressions of the two players at the bottom (i.e., the victim and one of the perpetrators in the Exclusion condition, and their counterparts in the Control condition) in terms of characteristics detailed below; the order of evaluations (victim first or perpetrator first) was counterbalanced. After completing a demographics questionnaire and an open-ended question about their guesses of the study’s real purpose, participants were thanked and fully debriefed.

### Measures

All questions were presented accompanied by 7-point Likert scales (e.g., 1 = “Not at all”; 7 = “Very much”).

#### Dehumanization

Participants rated the two players on a list of 20 traits of HN and HU (translated from Bastian and Haslam, 2010 into Korean by the authors) with 5 positive and 5 negative traits on each dimension. Participants were asked to “evaluate (the name of each player) on the characteristics she is expected to have, based on the game you just observed,” and then to “rate on the given scale how much (each trait word) describes (the name of each player).” Traits included “active,” “curious,” “friendly,” “helpful,” “fun-loving,” “impatient,” “impulsive,” “jealous,” “nervous,” and “shy” for HN, and “broadminded,” “conscientious,” “humble,” “polite,” and “thorough,” “disorganized,” “hard-hearted,” “ignorant,” “rude,” and “stingy” for HU (5 each for positive and negative dimensions). The traits were presented in a random order. Two positive (“friendly” and “helpful”) and two negative (“nervous” and “shy”) HN traits did not correlate well with other items in each scale, and were accordingly excluded from average scores (excluding them did not significantly change the results). Cronbach’s αs for the victim were 0.83 (positive) and 0.70 (negative) for HN traits, and 0.78 (positive) and 0.73 (negative) for HU traits. For the perpetrator, Cronbach’s α = 0.77 and 0.62 each for positive and negative HN traits, and 0.81 and 0.73 each for positive and negative HU traits. Initial examination of the relationships between HN and HU scores revealed different patterns depending on the valence: Positive HN and HU scores were either weakly correlated (for the victim, *r* = 0.21, *p* = 0.002) or uncorrelated (for the perpetrator, *r* = –0.05, ns), but the negative scores were strongly positively correlated (*r* = 0.63, *p* < 0.001 for the victim and *r* = 0.69, *p* < 0.001 for the perpetrator). That the negative HN and HU scores were strongly correlated with each other poses concerns over how to interpret results involving them; this issue is further examined in Discussion.

#### Vulnerability to Exploitation

To measure the proneness to be exploited by other people, we asked participants the question “How easily would the target person be taken advantage of, compared to other people?” The scores were recoded so that a higher score mean being more vulnerable to exploitation.

#### Agency and Experience

According to Gray et al. (2007), minds have two qualities, each of which varies on a continuum: Agency (the capacity to think and determine) and Experience (the capacity to feel emotions and senses). Targets who are perceived to be higher on Agency are more likely to be expected as agents of actions, while those viewed as higher on Experience are more likely to be seen as recipients (i.e., “patients; Gray and Wegner, 2009) of actions. Items measuring Agency and Experience (3 each) were adopted from Gray et al. (2007). Three items each that had the highest loadings on each factor in Gray et al.’s data were selected and used. Agency items (Cronbach’s αs = 0.85 and 0.83, respectively, for the victim and for the perpetrator) tapped mental capacities of self-control, morality, and memory (e.g., “How much is the target person capable of self-control, compared to other people?”). Experience items (Cronbach’s αs = 0.65 and 0.72, respectively, for the victim and for the perpetrator) evaluated the person’s susceptibility to feel pain, fear, and hunger (e.g., “How much is the target person likely to feel pain, compared to other people?”).

#### Intention/Responsibility

To see whether the victim and the perpetrator statuses would influence observers’ attribution of intention and responsibility for an action, we used 2 questions from Gray and Wegner (2009) that measure perceived intention and responsibility for a hypothetical, ambiguously harmful behavior. For each of the two targets, participants were asked to answer the questions “If X (the person’s name) bumped into someone who walked toward her from the opposite side, how intentional/responsible was the target’s behavior?” Because ratings for the two items were significantly correlated with each other (*r*s > 0.40, *p*s < 0.001), their mean scores were used for analysis.

#### Competence, Warmth, and Morality

In Fiske et al. (2007) Stereotype Content Model (SCM), Competence and Warmth are the two basic dimensions of human characteristics, which are ascribed to different extents to members of different social groups (Fiske et al., 2002). Those who are evaluated as low on these dimensions—especially those who are rated low on both—are perceived as less human. Leach et al. (2007) later argued for a tripartite model of social perception, adding Morality as a third, independent dimension. Items for these scales (3 traits each) were translated from those used in Leach et al. (2007): Competence (“intelligent”, “competent”, and “skilled”; Cronbach’s α = 0.78 and 0.75 respectively, for the victim and the perpetrator), Warmth (“likeable”, “warm”, and “friendly”; Cronbach’s αs = 0.86 and 0.84), and Morality (“honest”, “sincere”, and “trustworthy”; Cronbach’s αs = 0.82 and 0.77).

#### Personality Traits

Participants completed a shortened 15-item Korean version of big five inventory (BFI; John and Srivastava, 1999) translated and validated by Kim et al. (2011). Participants rated the two targets on Extraversion, Agreeableness, Conscientiousness, Neuroticism, and Openness to Experience, with 3 items each (e.g., “is talkative” for Extraversion; “has a vivid imagination” for Openness). All five factors showed high internal consistencies (all Cronbach’s αs at least 0.78).

## Results

### Humanness

For the sake of brevity, we will refer to the two targets in the Control condition as the victim and the perpetrator, based on their correspondence (i.e., same face image used) with those in the Exclusion condition. When corresponding targets in the two conditions are compared to each other, we will refer to those in the Control condition as *controls*.

**FIGURE 1:**
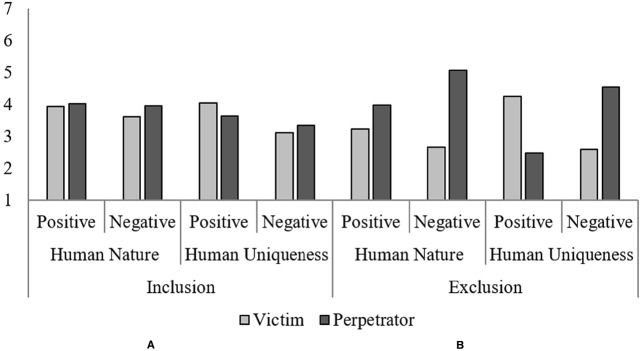
**Human Nature and Human Uniqueness scores.** The left side **(A)** and the right side **(B)** depict the Inclusion and Exclusion conditions, respectively.

Mean ratings of HN and HU were analyzed using three-way mixed ANOVAs with Player (Victim or Perpetrator) and Valence (Positive or Negative) as within-subject factors, and Condition (Exclusion or Control) as the between-subject factor. Table 1 lists the descriptive statistics of these measures (as well as all the other target perception measures) in different conditions, and Figures 1A,B depicts HN and HU scores broken down by the factors.

**TABLE 1:** **Means (standard deviations) of target perception measures**.

****	**Victim**		**Perpetrator**	
	**Exclusion**	**Inclusion**	**Exclusion**	**Inclusion**
***Human Nature***				
Positive	3.22 (1.27)	3.93 (1.16)	3.98 (1.44)	4.01 (1.23)
Negative	2.65 (1.01)	3.62 (1.06)	5.07 (1.19)	3.95 (1.13)
Human uniqueness				
Positive	4.24 (1.04)	4.04 (0.94)	2.48 (0.80)	3.63 (0.96)
Negative	2.60 (0.85)	3.10 (0.93)	4.54 (1.02)	3.33 (0.96)
				
Vulnerability to Exploitation	4.66 (1.43)	4.23 (1.36)	4.00 (1.80)	4.20 (1.50)
				
Agency	4.51 (0.79)	4.27 (0.95)	3.15 (0.92)	3.97 (1.07)
Experience	4.02 (0.94)	4.03 (0.80)	4.49 (1.23)	4.25 (0.85)
				
Intention/Responsibility	2.74 (1.07)	3.28 (1.11)	4.31 (1.08)	3.53 (1.21)
				
Competence	3.70 (1.04)	3.78 (0.97)	2.91 (1.04)	3.72 (0.92)
Warmth	4.03 (1.33)	3.81 (1.22)	2.32 (1.00)	3.54 (1.10)
Morality	4.05 (1.13)	4.08 (1.00)	3.19 (1.17)	3.88 (0.98)
				
Extraversion	3.01 (1.30)	3.81 (1.33)	4.17 (1.60)	3.98 (1.32)
Agreeableness	4.82 (1.11)	4.21 (1.31)	2.50 (1.07)	3.82 (1.16)
Conscientiousness	4.15 (1.15)	4.22 (0.99)	3.14 (1.11)	3.94 (1.09)
Neuroticism	3.88 (1.31)	3.61 (1.14)	3.41 (1.42)	3.50 (1.21)
Openness to experience	3.32 (1.15)	3.59 (1.11)	3.10 (1.33)	3.65 (1.13)

#### Human Nature

For HN traits, there was a significant main effects of Player, *F*(1, 216) = 85.14, *p* < 0.001, η^2^ = 0.28, as well as a marginally significant main effect of Condition, *F*(1, 216) = 2.79, *p* = 0.10, η^2^ = 0.01. There were tendencies of the victim being perceived as less human, as well as of players in the Exclusion condition being more dehumanized. Valence main effect was not significant. All two-way interactions were significant: Player × Valence, *F*(1, 216) = 40.89, *p* < 0.001, η^2^ = 0.16; Valence × Condition, *F*(1, 216) = 14.60, *p* < 0.001, η^2^ = 0.06; and more importantly, the expected Player × Condition interaction, *F*(1, 216) = 50.51, *p* < 0.001, η^2^ = 0.19. A closer examination of the last interaction revealed that the victim was rated as having less HN characteristics than her counterpart in the Control condition, *F*(1, 216) = 50.86, *p* < 0.001, η^2^ = 0.19, while the perpetrator was viewed as having more HN traits than the control, *F*(1, 216) = 14.57, *p* < 0.001, η^2^ = 0.06. The results support our hypothesis that the exclusion victim would be perceived as less human by observers, while also showing that the perpetrator of exclusion is seen to be *more* human than even someone uninvolved in exclusion. Lastly, these two-way interactions were qualified by a significant three-way interaction of Player × Valence × Condition, *F*(1, 216) = 22.68, *p* < 0.001, η^2^ = 0.10. Simple effects analyses demonstrated that the victim was attributed less HN characteristics on both positive and negative traits than the control; positive: *F*(1, 216) = 18.68, *p* < 0.001, η^2^ = 0.08, negative: *F*(1, 216) = 47.92, *p* < 0.001, η^2^ = 0.18. In other words, observers devalued the victim on both positive and negative aspects of HN. On the other hand, the perpetrator was attributed similar degrees of positive HN traits compared to the control but more negative HN traits; positive: *F*(1, 216) = 0.03, *ns*, negative: *F*(1, 216) = 50.85, *p* < 0.001, η^2^ = 0.19.

#### Human Uniqueness

For the HU dimension, the main effect of Valence was significant, *F*(1, 216) = 10.45, *p* = 0.001, η^2^ = 0.05 so that the targets were rated higher on positive than negative HU traits. Neither Player nor Condition main effects were significant, both *F*s < 1.01, *ns*. The expected Player × Condition interaction was significant, *F*(1, 216) = 3.91, *p* = 0.049, η^2^ = 0.02: Consistent with HN results, the victim was attributed marginally less HU traits than the control, *F*(1, 216) = 3.65, *p* = 0.06, η^2^ = 0.02. In contrast, no significant difference was found between the perpetrator and the control, *F*(1, 216) = 0.13, *ns*. Significant interactions of Player × Valence, *F*(1, 216) = 187.62, *p* < 0.001, η^2^ = 0.47, and of Valence × Condition, *F*(1, 216) = 42.56, *p* < 0.001, η^2^ = 0.17, were also observed. Again, these 2-way effects were qualified by a significant Player × Valence × Condition interaction, *F*(1, 216) = 93.50, *p* < 0.001, η^2^ = 0.30. Simple effects analyses revealed that the victim was comparable to the control in how much positive HU traits they were perceived to have, *F*(1, 216) = 2.13, *ns*, but was perceived to possess less negative HU traits compared to the control, *F*(1, 216) = 17.10, *p* < 0.001, η^2^ = 0.07. Meanwhile, the perpetrator was attributed less HU positive and more HU negative traits than the control; positive: *F*(1, 216) = 93.18, *p* < 0.001, η^2^ = 0.30, negative: *F*(1, 216) = 81.51, *p* < 0.001, η^2^ = 0.27.

Results of humanness ratings revealed that, first, the victim was perceived as having less HN traits and marginally less HU traits compared to the control. The difference in HN traits were driven both by exclusion effects on both positive and negative traits, while the difference in HU traits were driven mostly by exclusion effect on negative traits. In other words, the exclusion victim was dehumanized in terms of HN characteristics largely across the board, while HU-related dehumanization happened mainly with negative characteristics. These results—especially that regarding HN dimension—support our hypothesis that when people get excluded, they are perceived at a lower degree of humanness. The data also revealed that this dehumanizing effect was more prominent in the domain of HN than HU: When only the victim data was examined, the Condition × Humanness Dimensions (HN vs. HU) 2-way interaction effect was significant, *F*(1, 216) = 34.26, *p* < 0.001, η^2^ = 0.14, as was the Valence × Condition × Humanness Dimensions 3-way interaction effect, *F*(1, 216) = 4.64, *p* = 0.03, η^2^ = 0.02.

On the other hand, perception of exclusion perpetrators showed a different picture. Participants saw the perpetrator as even more human than the control on traits about HN, and this was mostly due to higher ratings on negative HN traits—no significant difference in positive HN traits were observed. For HU traits, ratings of the perpetrator were lower for positive traits but higher for negative traits; in other words, the pattern indicates that participants merely made a more negative evaluation of the perpetrator compared to the control, rather than humanizing (or dehumanizing) her in terms of HU.

### Vulnerability to Exploitation and Mediations

#### Vulnerability to Exploitation

There was a main effect of Player, *F*(1, 216) = 4.87, *p* = 0.03, η^2^ = 0.02, but not of Condition, *F*(1, 216) = 0.71, *ns*. Player × Condition interaction for Vulnerability to Exploitation was significant, *F*(1, 216) = 4.12, *p* = 0.04, η^2^ = 0.02, with simple effects demonstrating that the victim’s perceived likelihood of being taken advantage of was higher than that of the control, *F*(1, 216) = 8.97, *p* = 0.003, η^2^ = 0.04, but for the perpetrator no difference was observed, *F*(1, 216) = 0.02, *ns*. In other words, participants were more likely to expect that the victim would be at the mercy of others (Table 1), which is consistent with a popular image of an ostracism victim as a target of various kinds of social manipulations.

To test whether effects of social exclusion manipulation on perceived humanness of the victim and the perpetrator explain expectations of their Vulnerability to Exploitation, we ran parallel mediation analyses separately for the victim and for the perpetrator with Condition (Inclusion coded as –1 and Exclusion as 1) as the IV, two dimensions of humanness as the mediators, and Vulnerability to Exploitation as the DV using bootstrapping procedure with SPSS PROCESS macro by Hayes (2013) with 1,000 resamplings. For the victim, HN significantly mediated the relation between Condition and Vulnerability to Exploitation, *B* = 0.10 with 95% confidence interval (CI) of (0.00, 0.23); HU, on the other hand, did not mediate the relation significantly, *B* = –0.02, 95% CI (–0.07, 0.01). The mediating effect of HN on Condition-Vulnerability relation for the perpetrator was also significant, *B* = –0.05, 95% CI (–0.10, –0.01), but HU again did not exhibit such a mediating effect, *B* = –0.00, 95% CI (–0.04, 0.01). For both kinds of players, HN negatively predicted Vulnerability to Exploitation. This, coupled with higher assignment of HN for the exclusion victim (vs. the control and the perpetrator), seems to partly explain why observing a person being ostracized led to higher expectations of that person getting exploited by others—and the opposite for the perpetrator.

### Other Measures of Person Perception

Participants’ ratings of the targets on other measures were compared with two-way mixed ANOVAs, with Player as the within-subject factor and Condition as the between-subjects factor.

#### Agency and Experience

Both main effects of Player and of Condition on Agency were significant: *F*(1, 216) = 73.37, *p* < 0.001, η^2^ = 0.25 for Player and *F*(1, 216) = 12.67, *p* < 0.001, η^2^ = 0.06 for Condition. A significant Player × Condition interaction effect on Agency was also observed, *F*(1, 216) = 30.23, *p* < 0.001, η^2^ = 0.12, with simple effects of Condition and of Player showing that the victim’s perceived agency was *higher* than that of the control, *F*(1, 216) = 4.22, *p* = 0.04, η^2^ = 0.02, but the perpetrator was perceived as *less* agentic compared to the control, *F*(1, 216) = 37.32, *p* < 0.001, η^2^ = 0.15, as well as to the victim, *F*(1, 216) = 98.89, *p* < 0.001, η^2^ = 0.31. This was the opposite of what previous findings (Gray and Wegner, 2009) indicate: It turned out to be the victim, not the perpetrator, that was seen as more agentic. Regarding Experience, only the main effect of Player was significant, *F*(1, 216) = 11.13, *p* = 0.001, η^2^ = 0.05, which was driven by a *higher* expectation of Experience for the perpetrator than for the victim in the exclusion condition, *F*(1, 216) = 10.22, *p* = 0.002, η^2^ = 0.05. Taken together, the results involving Agency and Experience present a puzzling pattern: In contrast to what HN and HU patterns revealed, the victim was perceived to be more agentic and to have less capacity for experience than the perpetrator. Although Agency and Experience items were not as clearly valenced as HN and HU items, it was possible participants thought of Agency items as reflecting positive aspects (i.e., the three items were all about high-level mental faculties) and Experience items as probing negative aspects (the experiences were all negative ones, such as hunger). Thus, it may have been that participants engaged in victim enhancement and/or perpetrator derogation using these dimensions.

#### Intention/Responsibility

There was a significant Player main effect, *F*(1, 216) = 67.54, *p* < 0.001, η^2^ = 0.24, as well as a significant Player × Condition interaction effect, *F*(1, 216) = 35.30, *p* < 0.001, η^2^ = 0.14 on the composite score of Intention/Responsibility. Examination of simple effects indicated that the victim was viewed to have less intention and responsibility for the harmful incident compared to the control, *F*(1, 216) = 13.14, *p* < 0.001, η^2^ = 0.06, while the perpetrator was perceived as having more intention and responsibility compared to the control, *F*(1, 216) = 25.16, *p* < 0.001, η^2^ = 0.10. The findings suggest that the victim was attributed less capabilities of engaging in voluntary behavior (Gray and Wegner, 2009). At the same time, it was also possible that the victim was rather enhanced by less blame, whereas the perpetrator was derogated and attributed more blame; because the behavior was plainly negative in nature (harming another person as its consequence), it is unclear whether the effects were driven by victim derogation (in terms of less attribution of intent and responsibility in general), victim enhancement (in terms of less attribution of intent and responsibility for a negative behavior only), or both.

#### Competence, Warmth and Morality

For all three measures of Competence, Warmth, and Morality, all main and interaction effects involving Player and Condition were significant, all *F*s > 10.62, all *p*s < 0.01, η^2^ values ranging 0.05–0.26. Examination of simple effects indicated that the perpetrator was seen as less competent, less warm, and less moral compared to the control as well to the victim, all *F*s > 22.26, all *p*s < 0.001, all η^2^ values >0.10, while the victim and the control did not differ on any of these dimensions, all *F*s < 2.21, *ns*. This indicates that these measures were used for perpetrator derogation.

#### Big 5 Traits

For Extraversion, Agreeableness, and Conscientiousness, all main and interaction effects involving Player and Condition were significant, with all *F*s > 9.29, *p*s < 0.003, and η^2^ ranging from 0.04 to 0.35. For Neuroticism, only a main effect of Player was observed, *F*(1, 216) = 4.61, *p* = 0.03, η^2^ = 0.02. Lastly, there was only a significant Player by Condition interaction effect on Openness to Experience, *F*(1, 216) = 15.34, *p* < 0.001, η^2^ = 0.07. Simple effects analyses revealed that the victim was evaluated as less extraverted and more agreeable compared to the control, *F*s > 13.69, *p*s < 0.001, η^2^ at least 0.06, and also less extraverted, more agreeable, more conscientious, and more neurotic compared to the perpetrator, *F*s > 6.06, *p*s < = 0.02, η^2^ > = 0.03. In contrast, the perpetrator was seen as less agreeable, less conscientious, and less open to experience compared to the control, all *F*s > = 10.45, *p* < = 0.001, η^2^ > = 0.05. These data draw a picture of the victim as a socially inhibited but nice and principled individual, while the impression of the perpetrator appears to be that of a socially more skilled, but less friendly, more careless, and closed-minded person.

## Discussion

This study tested whether evaluations of individuals on aspects of human characteristics would vary as a function of involvement in a social exclusion (as a victim, as a perpetrator, or uninvolved). The findings supported our hypothesis that third party observers would view a victim of ostracism as less human on both dimensions of HN and of HU. In stark contrast, the perpetrator was assigned even more humanness on 3 out of 4 aspects (i.e., positive and negative HN traits, and negative HU traits) compared to the control. The victim was also more (and the perpetrator, less) expected to be taken advantage of by others. A mediation analysis showed that these differences in expected likelihood of being exploited can be explained by perception of less HN—but not HU—characteristics.

Overall, dehumanization as well as its mediating effect was more strongly observed on HN than on HU; this is in line with previous research (Haslam et al., 2005; Haslam and Bain, 2007; Bastian and Haslam, 2010). HN is the characteristics that humans are essentially born with and share with other people (Haslam et al., 2005), and being ostracized implies that one is expelled from the group she belonged to, i.e., the human race. Accordingly, HN should be the core dimension of humanness in the context of social disconnection and dehumanization, on which observers evaluate victims and perpetrators of ostracism to different degrees.

Negating the victim’s humanness has important implications for perception of their moral values. Those denied HN may be seen as undeserving of moral caring (Bastian et al., 2011) and would be treated out of moral boundary, which can result in observers’ indifference to the victim’s suffering or even active wrongdoing toward them. The victim and the harm inflicted on them threatens the observers’ need to maintain the belief about a just world (Lerner and Miller, 1978) or the need for feelings of protection (Shaver, 1970), and rationalizing an unjust situation by blaming the victim can be one of the strategies to restore the threatened motives (Lerner and Miller, 1978; Burger, 1981). Accordingly, to alleviate discomfort, observers may deploy victim blaming and derogation, by way of subtle denial of human qualities.

For most of the other human features examined, the overall results indicated that the victim was judged in an enhancing way whereas the perpetrator was perceived in a derogating way. Compared to the perpetrator, the victim was assessed as more agentic, more competent, warmer, more moral, more agreeable, and more conscientious. Although these results seem to go against findings on HN and HU, it is not entirely inconceivable that different aspects of impressions of an exclusion victim (and of a perpetrator) are at odds with one another. Kay et al. (2005) demonstrated that when people’s belief that the world is a just place is threatened by witnessing adversities of a victim, they would use both victim derogation and victim enhancement as functionally compatible means to maintain the belief. Additionally in Callan and Ellard’s (2003) study, people explicitly evaluated a victim in a positive way but implicitly devaluated the same person. In line with these studies, our finding confirmed that two opposite evaluations of a person (i.e., dehumanization and enhancement of the same victim) are possible, which suggests that behind favorable attitudes toward a victim, people can dehumanize her at the same time.

As mentioned in Materials and Methods, the negative HN and HU scores were correlated with each other. Accordingly, interpretations involving those scores should be made with caution. Given that the two dimensions reflect distinct aspects of mind perception (and therefore should be independent for each other), it is possible that participants only focused on the valence of the negative trait terms rather than differentiating the two. So instead of dehumanizing the victim relative to the perpetrator, it may have been that participants simply derogated the perpetrator. Although this is highly possible, it does not critically compromise our general claim because, after all, the main finding of interest involves the higher positive HN rating for the perpetrator. Because the positive HN and HU scores were only weakly correlated with each other, it is safer to assume that they indeed tap HN and HU.

Future research can investigate issues unexplored or unresolved in the current study. First, the results involving Agency and Experience are in conflict with previous research, which for example demonstrated that, by ascribing more Experience, people viewed the victim of date rape as a moral patient who is susceptible to harm and pain (Gray and Wegner, 2009). These inconsistent results about victims and perpetrators may be due to differences in the nature of what targets in different studies go through. Future research would need to test whether, depending on the kinds and intensities of harm inflicted on victims, judgments of victims’ humanness characteristics would show different patterns. Second, in our research, observers devalue the humanness of a person after watching strangers seemingly ostracize that person for a short period. Observers’ judgments of a target person may vary according to group membership of the person. People tend to consider more favorably, and act more generously to, ingroup than outgroup members (Dovidio and Gaertner, 2010). Future work could examine if observers’ evaluation of human values of a victim would differ depending on whether a victim is an ingroup or an outgroup member. Third, we argued that denying one’s humanness is a subtle way of derogating people that could be an underlying mechanism of justification of ostracism. Future research can more directly test the relationship between victim dehumanization and ostracism justification, by way of examining whether being a victim of social exclusion would render one less deserving of moral care. In addition, validity of the current findings could be bolstered using alternate methods to assess humanness attribution (such as implicit measurement; Loughnan and Haslam, 2007). One limitation of the current study involves measurement of humanness dimensions, namely that we had to remove some items because of internal consistency issues; replication using alternative methods would help addressing interpretation ambiguities borne out by such measurement problems.

In conclusion, our research presents evidence that from the perspective of third party observers, a victim of social exclusion can be seen as less human. In order to prevent or minimize damages to a victim, it is important to understand how observers perceive the victim, which can strongly influence observers’ attitude toward her (Sainio et al., 2011; Salmivalli et al., 2011). Yet to date there has been little work on perceived humanness of ostracism victims from a third party perspective. Our finding that people would deny a victim full humanity is alarming because, within everyday social interactions, this kind of devaluation on others can be commonly occurring. It implies that to others’ eyes, whether you are “in” or “out” partly decides whether or not you are fully human.

## Author Contributions

YOP was responsible for the original research question. YOP and SHP were responsible for study design, data analysis, and writing and final approval of this manuscript.

### Conflict of Interest Statement

The authors declare that the research was conducted in the absence of any commercial or financial relationships that could be construed as a potential conflict of interest.
